# Automated Medical Diagnosis of Alzheimer´s Disease Using an Efficient Net Convolutional Neural Network

**DOI:** 10.1007/s10916-023-01941-4

**Published:** 2023-05-02

**Authors:** Deevyankar Agarwal, Manuel Álvaro Berbís, Antonio Luna, Vivian Lipari, Julien Brito Ballester, Isabel de la Torre-Díez

**Affiliations:** 1 Department of Signal Theory and Communications and Telematics Engineering, University of Valladolid, Paseo de Belén 15, 47011 Valladolid, Spain; 2 Hospital San Juan de Dios, HT Médica, Avda Brillante 106, 14012 Córdoba, Spain; 3 MRI Unit, Radiology Department, HT Médica, Carmelo Torres No. 2, 23007 Jaén, Spain; 4 European Atlantic University, Isabel Torres 21, 39011 Santander, Spain

**Keywords:** Alzheimer´s disease, Convolutional neural network, Deep learning, EfficientNet, Mild cognitive impairment, MRI, MONAI, Transfer learning

## Abstract

Alzheimer's disease (AD) poses an enormous challenge to modern healthcare. Since 2017, researchers have been using deep learning (DL) models for the early detection of AD using neuroimaging biomarkers. In this paper, we implement the EfficietNet-b0 convolutional neural network (CNN) with a novel approach—"fusion of end-to-end and transfer learning"—to classify different stages of AD. 245 T1W MRI scans of cognitively normal (CN) subjects, 229 scans of AD subjects, and 229 scans of subjects with stable mild cognitive impairment (sMCI) were employed. Each scan was preprocessed using a standard pipeline. The proposed models were trained and evaluated using preprocessed scans. For the sMCI vs. AD classification task we obtained 95.29% accuracy and 95.35% area under the curve (AUC) for model training and 93.10% accuracy and 93.00% AUC for model testing. For the multiclass AD vs. CN vs. sMCI classification task we obtained 85.66% accuracy and 86% AUC for model training and 87.38% accuracy and 88.00% AUC for model testing. Based on our experimental results, we conclude that CNN-based DL models can be used to analyze complicated MRI scan features in clinical settings.

## Introduction

Alzheimer’s disease (AD), the most prevalent kind of dementia, has no recognized disease-modifying therapy to date. It is characterized by a silent onset, in which AD gradually advances over a number of years before any clinical signs appear [1]. At least fifty million people worldwide are thought to be affected by AD and other forms of dementia [2]. As of 2022, there are 7 million AD patients in the USA, and 14 million individuals are predicted to be affected by the disease by 2050 [3]. For various forms of dementia, such as AD or mild cognitive impairment (MCI), it is essential to investigate novel early diagnostic techniques to ensure proper treatment and halt the progression of the illness. Between healthy cognitive function and AD lies a condition known as MCI. A person with MCI presents cognitive impairment but is nevertheless capable of conducting daily activities. MCI affects around one-fifth of the population over 65 years old, and about one-third of them will develop AD within three to five years [3]. Subjects with MCI will either develop AD or remain stable. Only autopsy can certify an AD diagnosis [4]. Nonetheless, biological and functional brain problems linked to AD may be examined and assessed using magnetic resonance imaging (MRI), often utilized in clinical practice and recognized as a helpful technique to identify the course of AD [4, 5].

Computer-aided machine learning (ML) techniques offer a systematic means to create complex, automatic classification models to manage large amounts of data and can find intricate and subtle patterns. Support vector machines (SVMs), logistic regression (LR), and SVM-recursive feature elimination are ML pattern analysis techniques that have been proved successful in AD detection [6]. However, automated diagnostic ML models for neuropsychiatric disorders based on SVMs require hand-made features because they cannot pull out adaptive features [6]. When utilizing these ML approaches for classification, the architectural design must be established. In general, four phases are required: feature extraction, feature selection, dimension reduction, and implementation of a classification method. Additionally, this process must be optimized, and specialists need to be involved at every level [7]. The use of deep learning (DL) models to predict different AD stages was made possible by the growing GPU processing power. The field of ML known as DL simulates how the human brain finds complicated patterns. DL techniques like convolutional neural network (CNN) and sparse autoencoders [8–15] have recently surpassed statistical ML techniques. The use of CNNs has rapidly spread into a variety of domains, beginning with the outstanding performance of AlexNet in ImageNet large-scale visual recognition challenge [16] and then expanded into medical image analysis, starting with 2D images, such as chest X-rays [17], and then progressing onto 3D images, including MRI. *End-to-end learning* (E2EL) is the central principle of DL. A key advantage of E2EL is that it simultaneously improves every stage of the processing pipeline, potentially resulting in an optimum performance [18].

For the analysis of MRI scans, Oh et al*.* [19] suggested an *end-to-end hierarchy extending between 1 to 4 levels*. Feature selection and extraction are carried out manually at Level 1 [20, 21]. Level 2 involves either the segmentation of 3D data into ROI or their conversion into 2D slices, following by their use as input to train the DL model, that can be either self-designed DL architecture or a CNN-based *pertained transfer learning* (TL) architecture like ResNet[12], deep ResNet [15], CaffeNet [22], DenseNet [23], etc.

According to recent research TL, which allows successful DL training even with limited data, is becoming a quite popular area of DL [24, 25]. TL is compared to human behavior as it may use the acquired information to solve new challenging situations.

When comparing stable MCI (sMCI) vs. progressive MCI (pMCI) or AD vs. sMCI classifications, it is simple enough to classify AD vs. CN, as there is a clear difference between the brain anatomy of AD and CN subjects and a sufficient availability of MRI scans [26]. Numerous studies [22, 27, 28] have also used *local TL* as a basis for this assumption. Local TL implies the classification of (sMCI, pMCI) or (sMCI, AD) subjects using the learning of the classifier that has been implemented to categorize (AD, CN) subjects. However, no studies using 3D pertained TL architecture have been found.

Preprocessed 3D MRI scans are fed into DL networks at Level 3. MRI scans must be preprocessed for any analytical analysis method to be effective [29]. During preprocessing, methods for noise removal, inhomogeneity correction, brain extraction, registration, leveling, and flattening are used to improve the quality of the image and make the architecture and brightness patterns consistent. A 3D MRI scan from the device is directly relayed as input into DL networks at Level 4; however, the authors are not aware of any studies utilizing this level.

Most of the *published empirical studies use either Level 1 or Level 2 learning*, their performance strongly relying on specific software and sometimes even on manual noise reduction and hyper parameter setup. As a result, only a subset of the original datasets were used for performance assessment, avoiding apparent outliers and making it difficult to fairly compare performances.

Mehmood et al*.* extracted gray matter (GM) tissue from MRI scans and fed it into the VGG-19 architecture to classify different AD phases [30]. A multi-class categorization of AD and its related stages using rs-fMRI and ResNet18 was done by Ramzan et al*.* [31]. During preprocessing, several studies [15, 32] segment the GM area, which is there after used as input for CNNs. Through the use of 3D-stacked Convolutional Autoencoders and MRI data, Hosseini-Asl et al*.* [33], by utilizing a Level 3 approach, reported the first effective use of a volumetric CNN-based architecture to classify AD and CN subjects.

Multimodal DL techniques [34–36] combine inputs from different data sources to better understand the structure and function of the brain by using several biological and physical properties to boost the classification accuracy of AD stages. Due to the limitations of the different resolutions, sheer number of dimensions, heterogeneous data, and limited sample sizes, multimodal DL techniques are particularly difficult to deploy at Level 3 learning [37]. In addition, we found that studies utilizing Level 2 learning mainly employed multimodal DL approaches. A unique deep neural network (DNN)-based approach was presented by Lu et al*.* [38] with multi modalities FDG-PET and T1-MRI to classify sMCI and pMCI subjects.Song et al*.* [39] created the "GM-PET" fused modality by combining the GM tissue region of MRI scans with FDG-PET images utilizing mask encoding and registration to aid in the diagnosis of AD. Only a small number of investigations utilized Level 3 architecture. The AD-NET was presented by Gao et al*.* [28], with the pre-training model performing the dual functions of extracting and transferring features as well as learning and transferring information. VoxCNN, based on deep 3D CNN, was proposed by Korolev et al*.* [40] for AD early detection. A multimodal DL framework based on multi-task CNN and 3D DenseNet was proposed by Liu et al*.* [41] for concurrent hippocampus segmentation and AD classification.

Literature demonstrates that *early detection of AD is essential for the patient to obtain maximum benefit*. As of right now, the highest accuracy level for this task utilizing either E2EL, local TL, or a CNN-based 2D transfer learning or ROI segmentation strategy is *86.30%* [42]. Therefore, *to enhance accuracy as well as the generalization capability of the model, we propose a fusion of E2EL and TL during the training phase of the model*. We trained the EfficientNet-b0 CNN for the AD vs. sMCI binary classification task by transferring the learning of each fold of fivefold stratified cross-validation to its subsequent fold, and so on. In the first fold, the model was trained using E2EL. We also trained and evaluated an E2EL-based EfficientNet-b0 model for the multiclass AD vs. CN vs. sMCI classification task.

Preprocessed 3D T1W MRI scans were fed into the models. *ANTsPyNet* [43] was used to preprocess MRI scans, whereas the Medical Open Network for Artificial Intelligence *(MONAI)* was used to design and implement the models [44]. The entire implementation was done in PyTorch GPU utilizing Google CoLab Pro + . To conclude, the following are the *main contributions* made by this article:

- We propose a novel TL and E2EL fusion method to train DL models.
- We preprocessed MRI scans using ANTsPyNet.
- We implemented an EfficientNet-b0 CNN by using Level 3 learning and preprocessed MRI scans, to classify different AD stages.

The rest of this article is structured as follows: research methodology, datasets, input management, DL models, and experimental setup are described in “Materials & methods” section, followed by results and discussion in “Experimental setup” section, and conclusions in “Results and discussion” section.

## Materials & methods

**Fig. 1:**
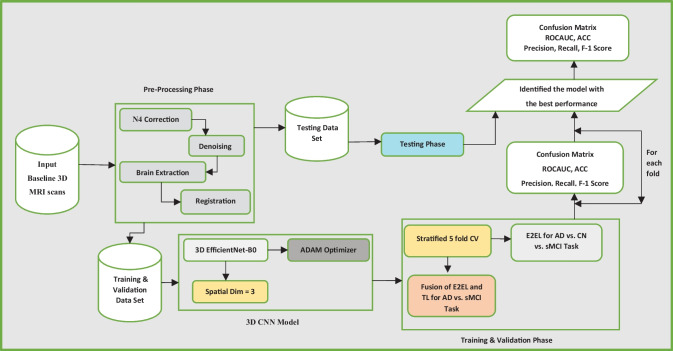
Block diagram of proposed work

We propose DL models for the classification of MRI scans of 1) AD and sMCI subjects, and 2) AD, sMCI, and CN subjects. For task one we used a novel E2EL and TL fusion approach, as shown in Fig. 1. During model training via fivefold stratified cross-validation, we trained the model from scratch in the first fold (E2EL), validated it, and used the final weights of the best epoch from fold 1 as the initial weights for fold 2 (TL). After training and validating the model in fold 2, we used the final weights of the best epoch from fold 2 as the initial weight for fold 3, and we repeated the same steps for the subsequent folds. For task 2, we used E2EL for all the folds. The model of the best epoch from each fold was used to assess external MRI scans to check for overfitting.

### Participants

We used the Information eXtraction from Images (IXI) [45] and Alzheimer's Disease Neuroimaging Initiative (ADNI) [46] databases, both of which are freely available online. The goal of ADNI is to identify biomarkers that may be used to monitor the course of AD as well as more sensitive and exact approaches for early AD detection. IXI is a collection of more than 600 MRI scans of healthy, normal individuals. Participants from different hospitals in London are included in the IXI dataset.

One of the most widely used sequences for structural brain imaging in clinical and research settings is the 3D magnetization-prepared rapid gradient-echo (MP-RAGE) sequence [47]. In a brief time, the sequence captures good tissue contrast and offers great spatial resolution with coverage of the whole brain. T1-weighted (T1W) sequences, which are a component of MRI, are thought of as “the most anatomical” of scans. These sequences provide the most accurate representation of tissues like white matter (WM), GM, and cerebrospinal fluid (CSF) [48]. NIfTI files are a kind of neuroimaging file format often utilized in image analytics in neuroscience and neuroradiology research.

We used 458 MP-RAGE T1W MRIs, 229 of AD subjects and 229 of sMCI subjects, all acquired in NIfTI format from ADNI. Only MCI scans that had been proven stable for at least four years and up to fifteen years were used. The remainder 245 scans of CN subjects were acquired from the IXI database.

### Preprocessing pipeline

**Fig. 2:**
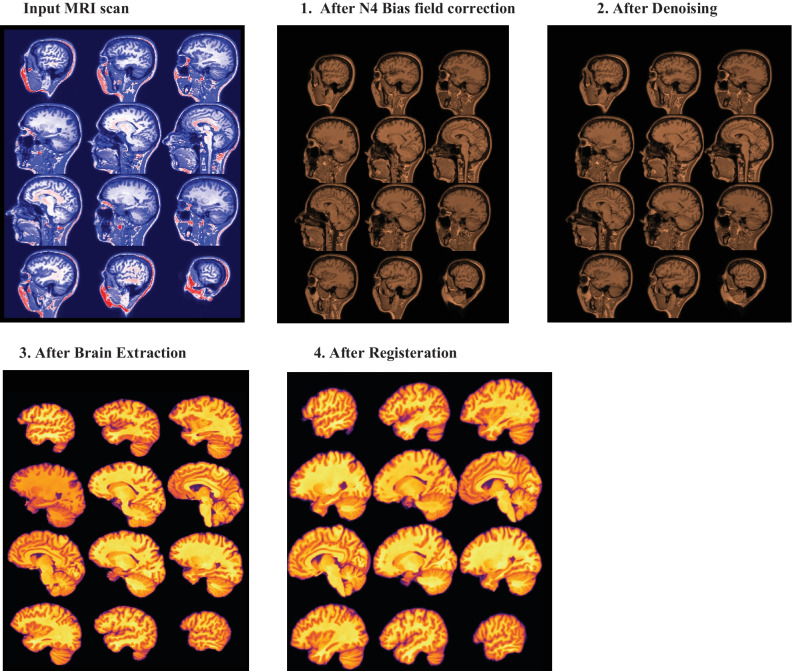
Output of each stage of the preprocessing pipeline for a cognitively normal subject {1➔2➔3➔4}. Input scan Dimensions: (256, 256, 150) Spacing: (0.9375, 0.9375, 1.2) Origin: (88.6399, -116.532, -112.1136). Output scan Dimension: (182, 218, 182) Spacing: (1.0, 1.0, 1.0) Origin: (-90.0, 126.0, -72.0)

Each image underwent a common preprocessing procedure using the ANtsPyNet [43] packages. The pipeline for Advanced Normalization Tools [49] was used. As shown in the Algorithm 1, a preprocessing pipeline was implemented for all MRI scans. Figure 2 depict the output of each stage for a CN subject with the corresponding dimension, spacing, and origin. After preprocessing, the dimensions after the final stage changed to 182 × 218 × 182 from the original image dimensions of 256 × 256 × 150.In the anatomical coordinate system, the origin is where the first voxel is located, and the spacing describes how far apart the voxels are along each axis. According to the MNI152 template specification, the spacing and origin values for all MRI scans also changed to (1.0, 1.0, 1.0) and (-90.0, 126.0, -72.0) respectively.

The *preprocessing procedure comprises the following parallel processes:* N4 bias field correction [50] to rectify the ferocity of a low-frequency signal irregularity, often known as bias field; Denoising [51] using a spatially adaptive filter to eliminate impulse noise; brain extraction [52] using a pertained U-Net model to remove non-brain tissues such as those found in the neck and skull; and registration to minimize the effects of transformations relative to a reference orientation as well as any spatial discrepancies across participants in the scanner. This procedure improved the accuracy of the classification. We affinely registered MRI scans to the MNI152 brain template [53], which was created by averaging 152 structural images into one large image using non-linear registration. The whole procedure to preprocess one MRI scan takes around three minutes.

**Algorithm 1:**
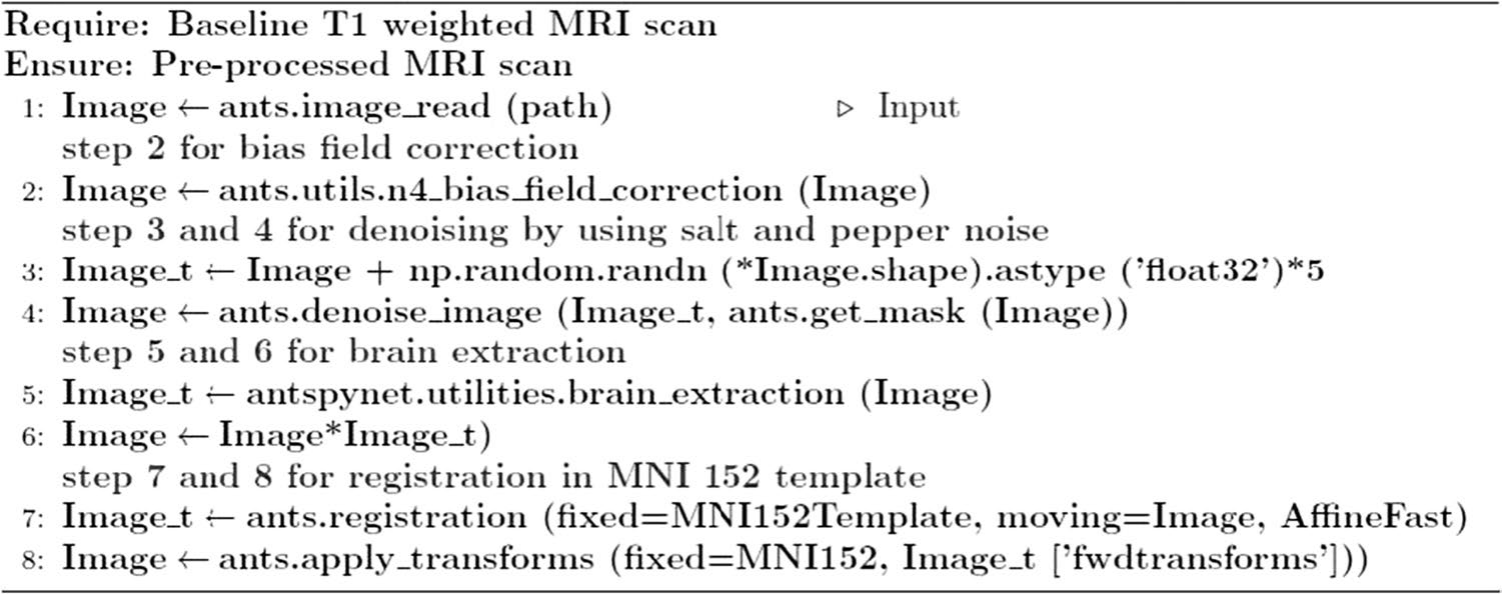
MRI pre-processing pipeline

### Implemented CNN

In recent years, CNNs have seen a surge in popularity because of their impressive usefulness in high-dimensional data analysis. EfficientNet models are based on simple and incredibly effective compound scaling methods. In many cases, EfficientNet models achieve better accuracy and efficiency than state-of-the-art CNNs like AlexNet, ImageNet, GoogleNet, or MobileNetV2 [17]. EfficientNets are more compact, run faster, and generalize more effectively, leading to improved accuracy. They have often been used with TL. However, they have only been pre-trained on 2D images, so their learning cannot be transferred to 3D MRI scans. Nonetheless, they can be trained for 3D scans via E2EL. Models from b0 to b7 [54] are represented in EfficientNet, with individual parameter sets spanning from 4.6 to 66 million. We chose the EfficientNet-B0 model for the proposed classification tasks because it offered the best overall evaluation metrics and the lowest model parameters, as reported by Agarwal et al. [55] in their implementation and comparative analysis of eight different CNNs for early detection of AD.

**Fig. 3:**
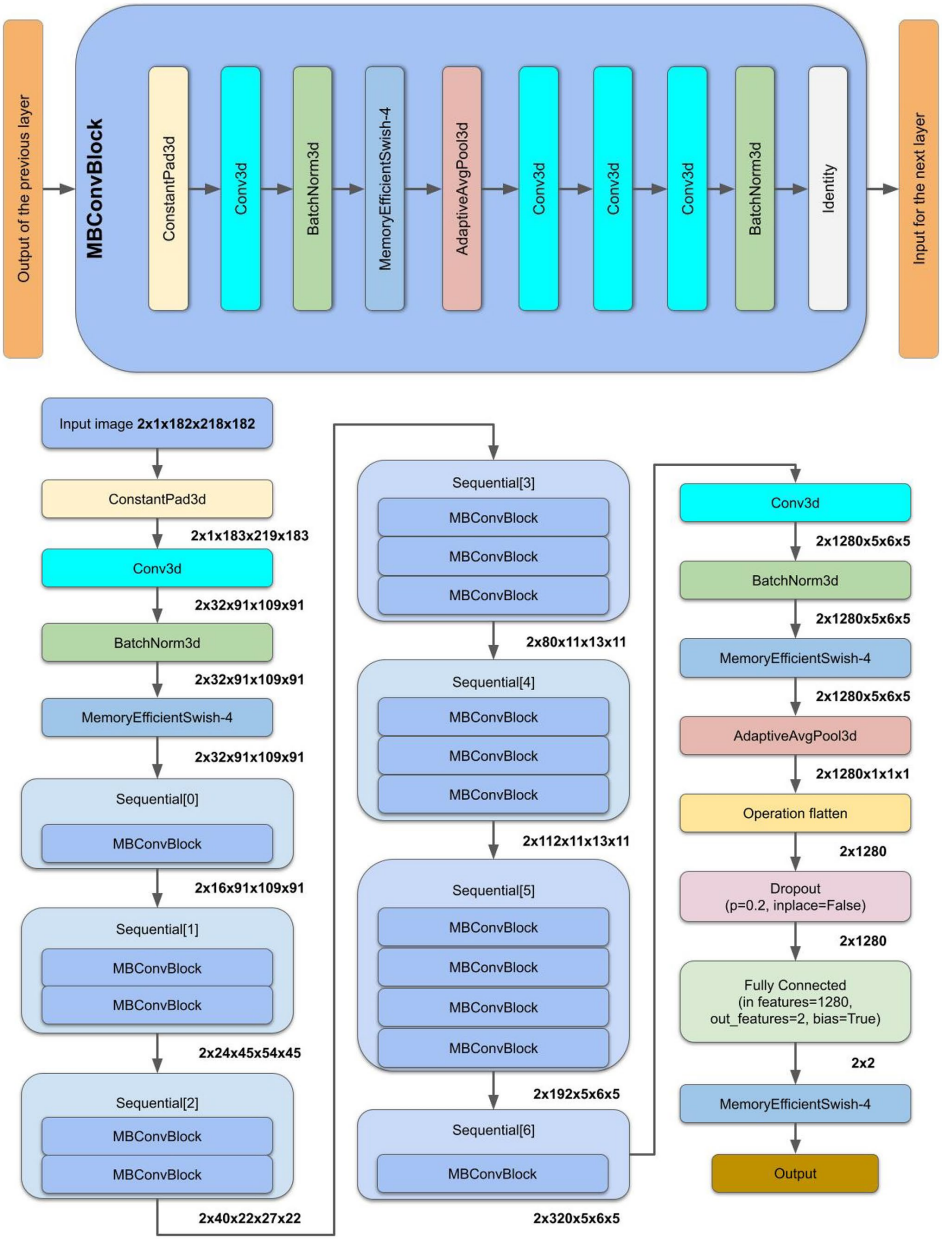
Structural layout of EfficienNet-B0

Figure 3 depicts the implemented EfficientNet-b0 structural layout. It has a total of 295 layers, distributed as shown in Table 1. Six consecutive blocks with various structures are included, in addition to 16 MBConvBlocks.

**Table 1:** Summary of the 295 layers of the model with output shape

**Input size**	**[2,1,182,218,182]**
**Layer (Type)**	**Output Shape**
ConstantPad3d	[2, 1, 183, 219, 183]
Conv3d	[2, 32, 91, 109, 91]
BatchNorm3d	[2, 32, 91, 109, 91]
MemoryEfficientSwish-4	[2, 32, 91, 109, 91]
Sequential [0] block Contains one MBConvBlock that contains following layers [ConstantPad3d,Conv3d, BatchNorm3d, MemoryEfficientSwish-4,AdaptiveAvgPool3d,again have three Conv3d and one BatchNorm3d,Identity]	[2, 16, 91, 109, 91]
Sequential [1] block contains two MBConvBlocks	[2, 24, 45, 54]
Sequential [2] block contains two MBConvBlocks	[2, 22, 27, 40]
Sequential [3] block contains three MBConvBlocks	[2,80,11,13,11]
Sequential [4] block contains three MBConvBlocks	[2,112,11,13,11]
Sequential [5] block contains four MBConvBlocks	[2,192,5,6,5]
Sequential [6] block contains one MBConvBlocks	[2,320,5,6,5]
Conv3d	[2,1280,5,6,5]
BatchNorm3d	[2,1280,5,6,5]
MemoryEfficientSwish-4	[2,1280,5,6,5]
AdaptiveAvgPool3d	[2,1280,1,1,1]
Operation flatten	[2,1280]
Dropout(*p* = 0.2, inplace = False)	[2,1280]
Fully Connected: Linear(in features = 1280, out_features = 2, bias = True)	[2]
MemoryEfficientSwish()	Output
**Total params: 4,690,942** **Trainable params: 4,690,942** **Non-trainable params: 0**	
**Input size (MB): 27.55** **Forward/backward pass size (MB): 7754.59** **Params size (MB): 17.89** **Estimated Total Size (MB): 7800.03**	

## Experimental setup

The whole implementation was completed using Google Colab Pro + [56], which was made available to the public in August 2021. Some of its most notable features include the ability to run in the background, early access to more powerful GPUs, and increased memory availability. Asynchronous data loading and multiprocessing are facilitated by GPUs. Despite not guaranteeing compatibility with a specific GPU, Colab Pro + does offer priority on the available options. Even with Pro + , GPU quality might decline after periods of intensive use. Pro + offers Tesla V100 or P100 NVIDIA Deep Learning GPU with CUDA support. The "High-RAM" option of Colab runtime met its objective by providing 52.8 GB RAM. Runtime support is supposed to be 24 h as stated in Colab's specs, yet we only got assistance for a maximum of 8 h. For this reason, we could not run all the folds at the same time in this setup, as finishing a fold with 50 epochs requires approximately 2 h. To implement a fivefold, stratified cross-validation, we first created five data sets (DATASETS 1–5) for training and validation, with the same class ratio as the original dataset across all folds. To train and validate Fold [n], we utilized Dataset [n]. The MRI scans were distributed as follows:

- AD: 160 for training, 40 for validation, 29 for testing.
- sMCI: 160 for training, 40 for validation, 29 for testing.
- CN: 160 for training, 40 for validation, 45 for testing.

**Fig. 4:**
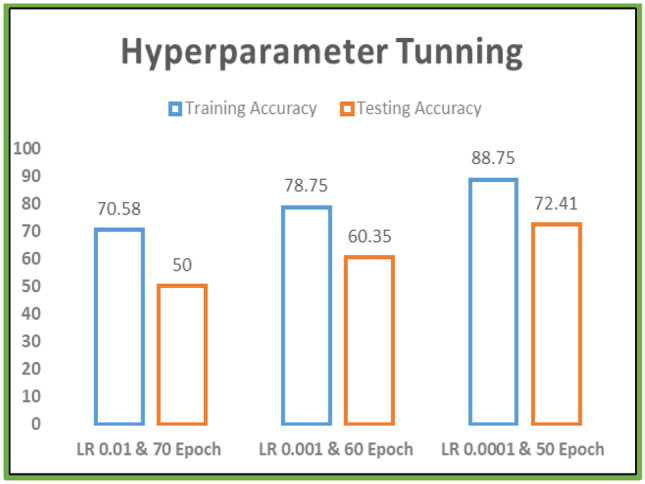
Tuning of the learning rate and number of epochs using a random search procedure and performance evaluation

All scans were prepared according to the preprocessing workflow shown in Fig. 2. Following preprocessing, five datasets were constructed and used, respectively, in folds 1–5. All models were implemented using MONAI, an open-source, PyTorch-based DL healthcare imaging platform maintained by the community. MONAI provides built-in PyTorch support along with a set of core features suited for the medical imaging area. Two important hyperparameters, learning rate and number of epochs, were optimized using the random search technique. We trained and tested the model on three different combinations of learning rate and number of epochs and, based on the comparison of their performances as shown in Fig. 4, learning rate was adjusted to 0.0001 and number of epochs to 50 for the final fivefold stratified CV implementation.

Adam is the first widely used "adaptive optimizer." It was used with a learning rate of 0.0001 and a batch size of 2. In the experiment, most cases converged after approximately 50 epochs. In addition, we used the cross-entropy loss function and the area under the curve for receiver operating characteristics (ROCAUC) metric to optimize the model weights during training and to evaluate the discriminatory abilities of the model across classes. The implemented method for the classification of sMCI vs. AD is presented in Algorithm 2. As indicated in steps 2 and 3, we utilized MRI scans from DATASET [1] for training and validation. In steps 48 and 49, we initialized the training and validation loaders from DATSET [C] MRI scans to be analyzed in fold C.

**Algorithm 2:**
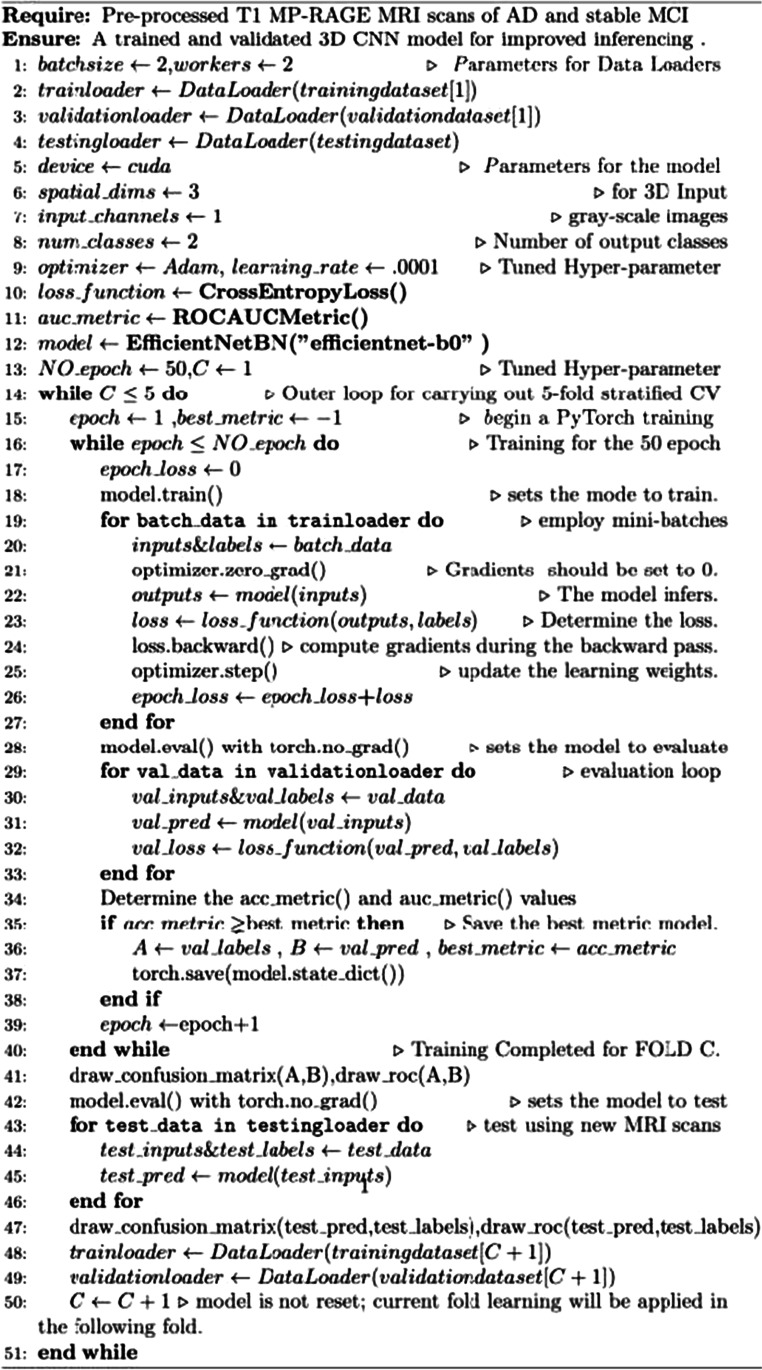
For sMCI vs. AD classification task

In steps 43 to 46, we evaluated unseen MRI scans using the model with the best weights from each fold. *We did not reset the model in step 50; conversely, we raised the counter to reflect the fold's rise since we intended to apply this fold's learning to the next fold*. The *same approach*, with a few modifications, was applied to the *AD, sMCI, and CN multiclassification task:* i.e., there were three types of *input MRI scans*; the *batch size and worker values were set to eight*; the number of output classes was changed to three; and the number of epochs was increased to 100. The same process was used for testing, validation, and training inside each fold. After each fold, the *model was reset to apply E2EL*.

The research training outcomes were evaluated using the following five metrics, as they provide critical data for the thorough evaluation of models that have been put into use: True Positive (TP), True Negative (TN), False Positive (FP), and False Negative (FN). Here, an AD patient is classified as TP or FN depending on whether they are placed in the AD group or not. Similarly, TN indicates the total number of individuals classified as presenting sMCI, and FP represents the total number of subjects not in the sMCI category. Accuracy (ACC) was defined as (TP + TN)/(TP + TN + FN + FP), while precision was defined as TP/(TP + FP). The positive sample prediction is more accurate when the precision is higher. Recall was defined as TP/(TP + FN), and the higher the recall rate, the more accurately the target sample may be predicted, and the less probable it is that a problematic sample will be missed. Precision and recall are often at odds with one another; hence, the F1-score is given as a composite statistic to balance their effects and evaluate classifiers more correctly. ROCAUC serves as a statistic to assess the ability of a model to distinguish between two classes. The effectiveness of the classification approach increases with the area under the ROC curve.

## Results and discussion

### Results

**Fig. 5:**
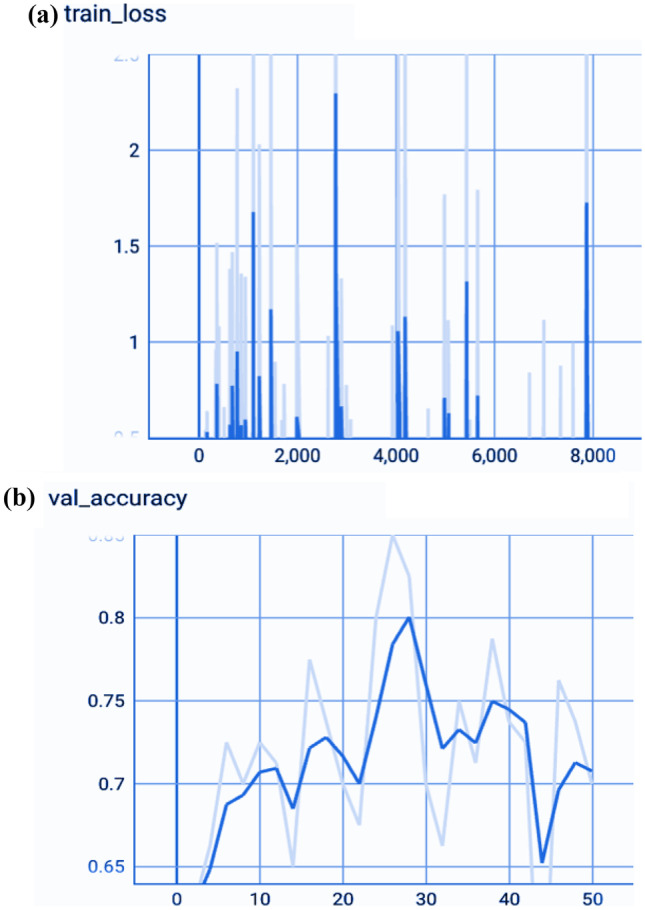
(**a**) Training loss of AD vs. sMCI task for fold 1 [Y axis: Training loss; X axis: Steps,160 batches * 50 Epochs = 8 K], (**b**) Validation Accuracy of AD vs. sMCI task for fold 1[Y axis: Validation accuracy; X axis: 50 Epochs]

**Fig. 6:**
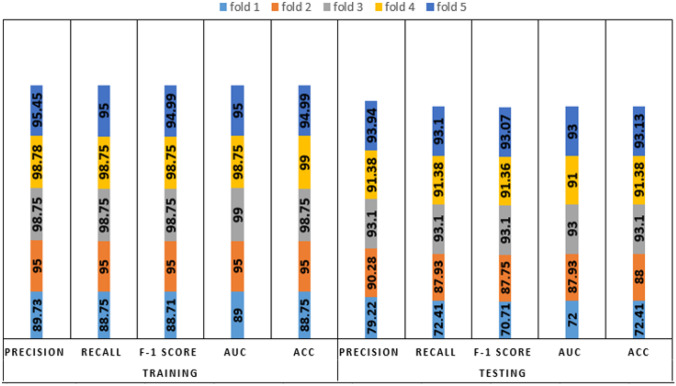
Comprehensive results for the AD vs. sMCI task

**Fig. 7:**
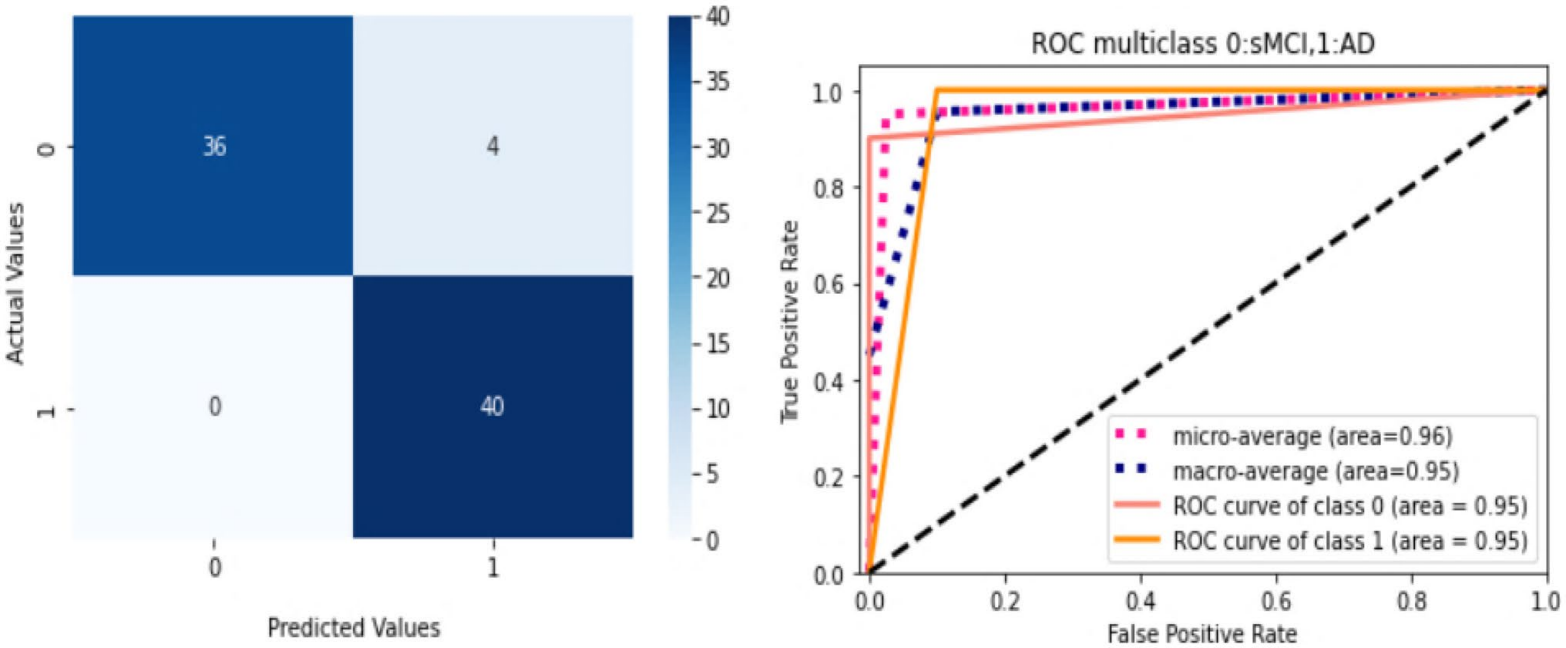
ROCAUC curve and the Confusion Matrix [0-sMCI,1-AD] for the optimal training fold for the AD vs. sMCI task

**Fig. 8:**
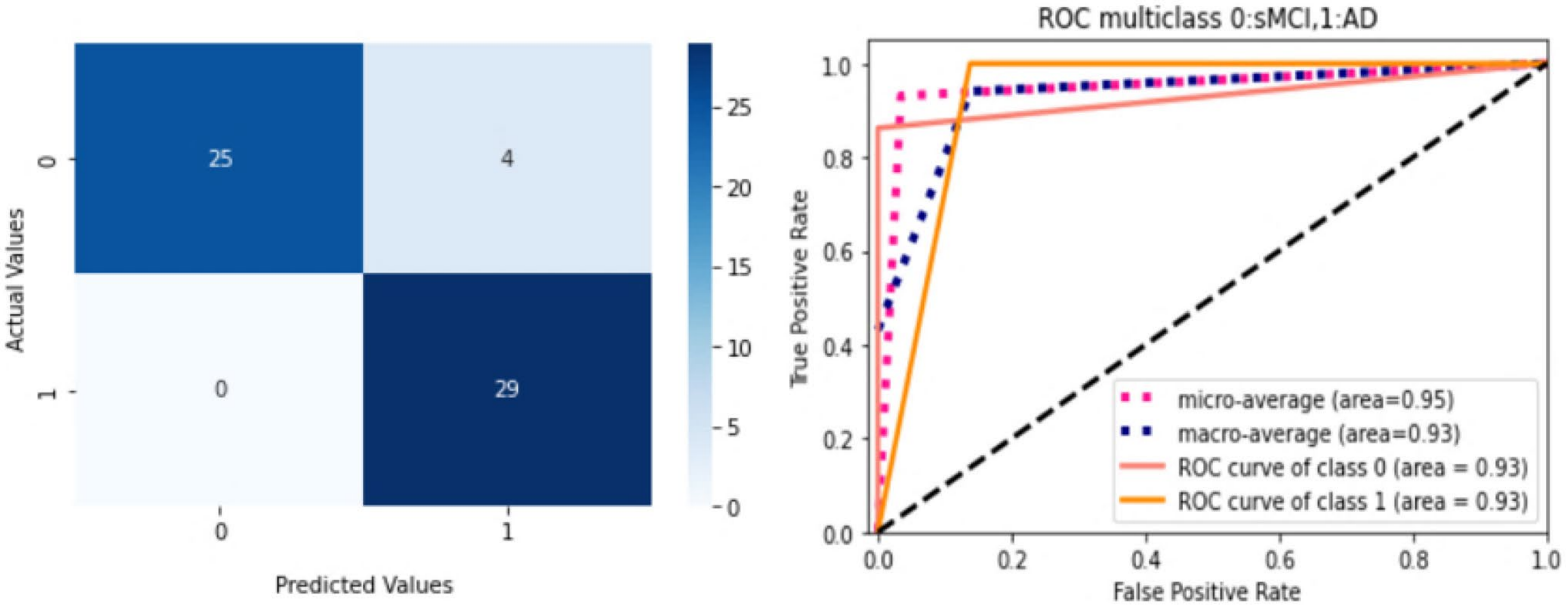
ROCAUC curve and the Confusion Matrix [0-sMCI,1-AD] for the optimal testing fold for the AD vs. sMCI task

We analyzed the performance of EfficientNet-B0 in the proposed tasks with the aim to understand its potential. Figure 5 depicts the loss experienced during training as well as the changes in validation accuracy of fold 1 for the AD vs. sMCI task. Training loss was progressively reduced while validation accuracy peaked at 88.75% in the first fold, indicating that the model was learning adequately. This effect increased in succeeding folds because of the application of transfer learning from prior folds, as can be seen in Fig. 6. Maximum testing accuracy and AUC reached 93.10% and 93.0% in fold 5, as shown in Fig. 6, while average training accuracy over all folds reached 95.29%. The confusion matrix and ROCAUC for the *optimal training and testing fold* of the binary classification task are shown in Figs. 7 and 8, respectively. A confusion matrix (an X-by-X matrix where X is the number of class labels) allows the assessment of the efficacy of a classification model. The matrix evaluates the accuracy of the predictions of the DL model vs. the actual target values. Accuracy, precision, recall, and F1-score may all be calculated via a confusion matrix. Validation was performed on a total of 80 MRI scans, with 40 scans utilized for each class. As shown in Fig. 7, TP for AD subjects was 40 and FN was 0, while TN for sMCI subjects was 36 and FP was 4. Due to the lack of information in the ADNI database regarding the status of each subject after x years of stability and as they present almost the same anatomical structures as AD scans, only a small number of sMCI subjects were misclassified. Both classes obtained an AUC of 95.0%.

Testing was performed on a total of 58 MRI scans (29 of AD and 29 of sMCI subjects) which had not been used during the training or validation procedures. As shown in Fig. 8, TP for AD subjects was 29 and FN was 0, while TN for sMCI subjects was 25 and FP was 4. Both classes obtained an AUC of 93.0%.

**Fig. 9:**
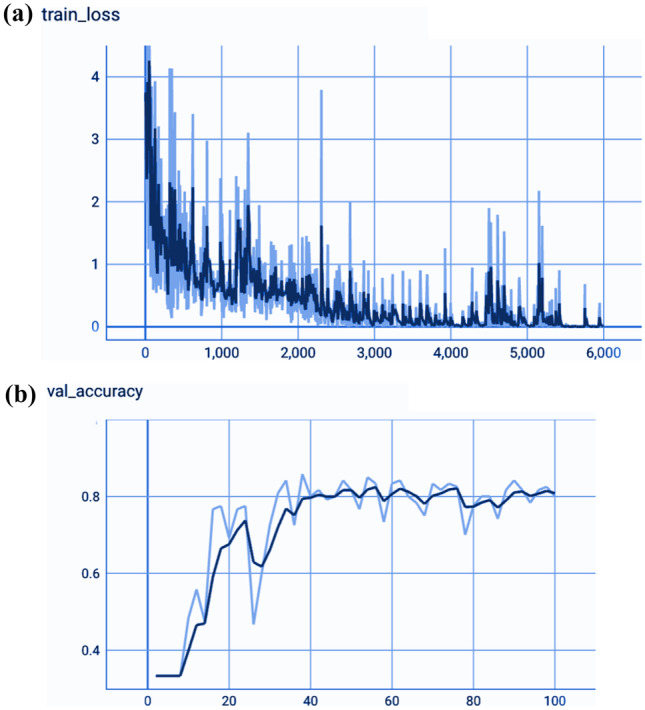
(**a**). Training loss of AD vs. sMCI vs. CN task for fold 1[Y axis: Training loss; X axis: Steps,60 batches * 100 Epochs = 6 K], (**b**). Validation Accuracy of AD vs. sMCI vs. CN task for fold 1 [Y axis: Validation accuracy; X axis: 100 Epochs]

**Fig. 10:**
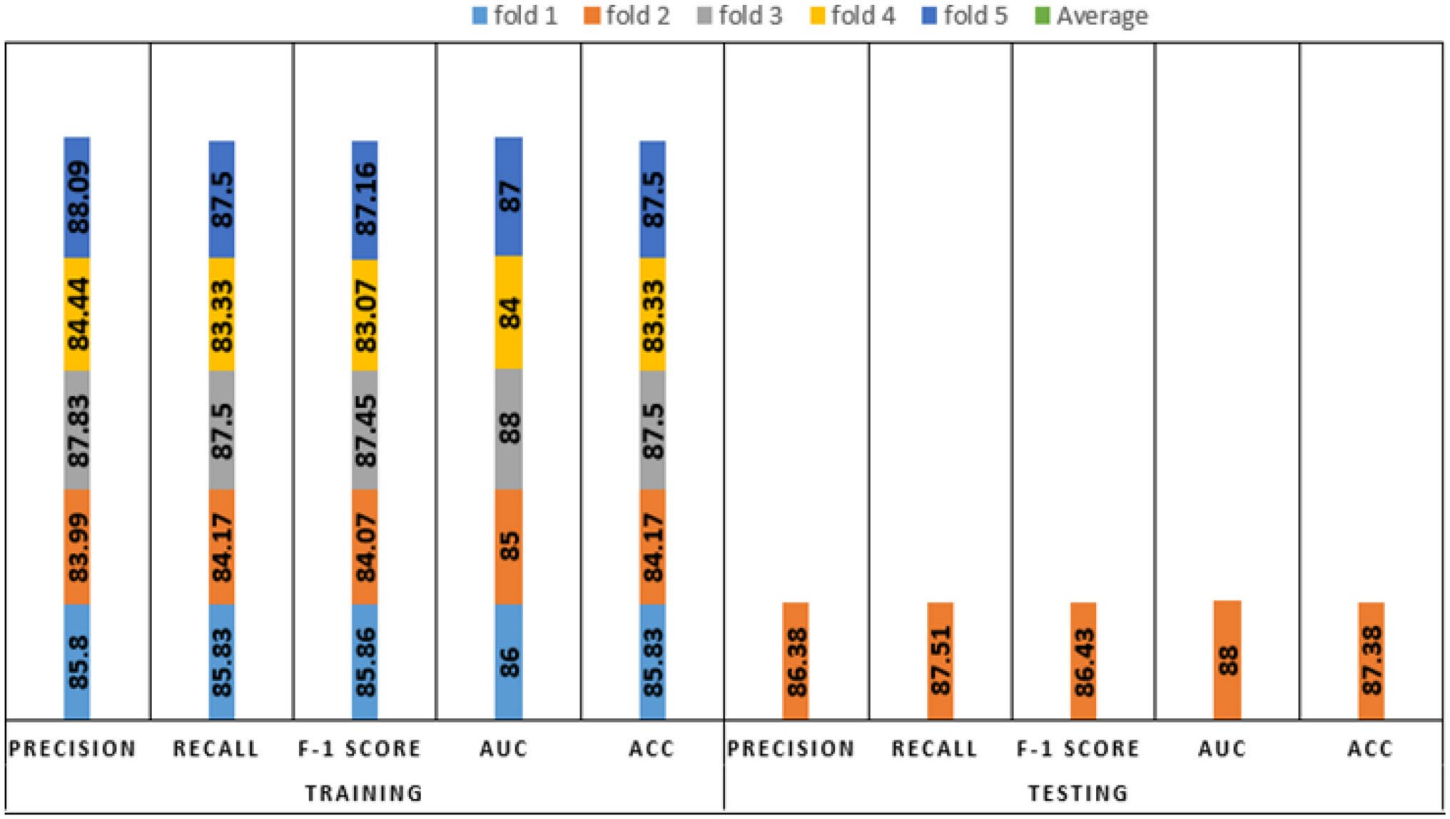
Comprehensive results for the AD vs. CN vs. sMCI task

**Fig. 11:**
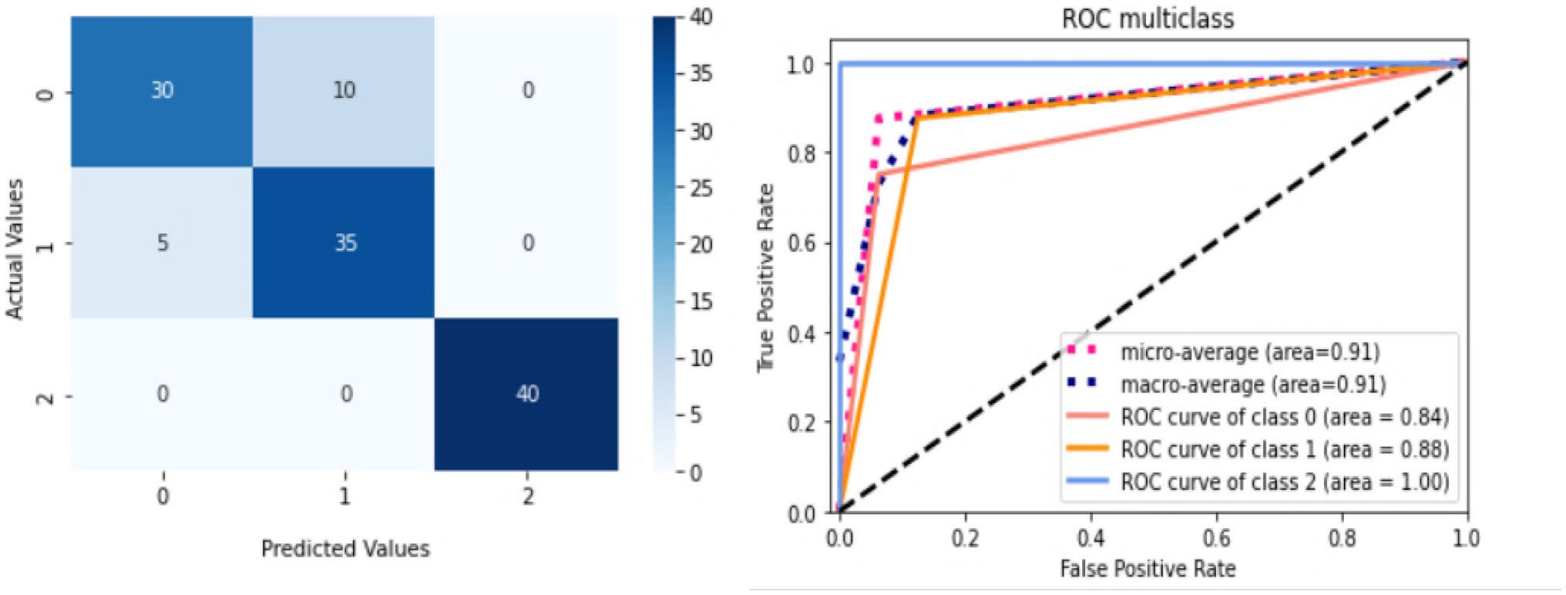
ROCAUC curve and the Confusion Matrix [0-sMCI,1-AD,2-CN] for the optimal training fold for the AD vs. CN vs. sMCI task

**Fig. 12:**
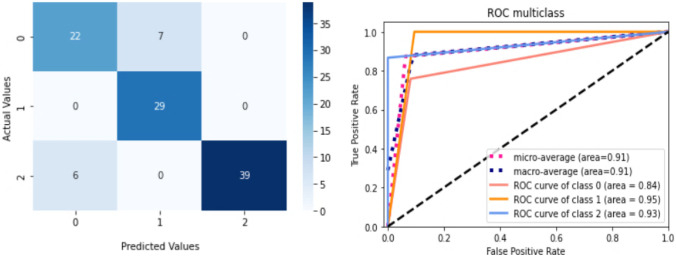
ROCAUC curve and the Confusion Matrix [0-sMCI,1-AD,2-CN] for the optimal testing fold for the AD vs. CN vs. sMCI task

Figure 9 depicts the training loss as well as variations in validation accuracy for the AD vs. sMCI vs. CN task. Training loss was progressively reduced, and validation accuracy peaked at 85.83% in the first fold, implying that the model was learning appropriately. As shown in Fig. 10, accuracy is altered in the subsequent folds due to learning from scratch in every fold. In fold 2, the highest testing accuracy reached 87.38%, while average training accuracy across all folds reached 85.66%. The confusion matrix and ROCAUC for the optimal training and testing fold of the multiclassification task are shown in Figs. 11 and 12, respectively. Validation was performed on a total of 120 MRI scans, with 40 scans utilized for each class. As shown in Fig. 11, nearly 100% accuracy was achieved for CN subjects; however, because of their similar anatomical structures, the categorization findings for the AD and sMCI participants indicated a small number of incorrect classifications.

The corresponding AUCs for the CN, AD, and sMCI classes were 100.0%, 88.0%, and 84%, respectively. Testing was performed on a total of 103 MRI scans (29 of AD, 29 of sMCI, and 45 of CN subjects) which had not been used during training or validation. As shown in Fig. 12, several sMCI and CN participants were incorrectly classified, while AD subjects were 100% correctly classified. The corresponding AUCs for the CN, AD, and sMCI classes were 93.0%, 95.0%, and 84%, respectively.

### Heat map visualization through occlusion sensitivity

Occlusion sensitivity is a simple way to figure out which parts of an image are most important for a deep network to classify [57]. Using small data tweaks, we may test a network's susceptibility to occlusion in various parts of the data. We utilize occlusion sensitivity to acquire high-level knowledge of what MRI scan attributes models employ to create a certain classification. The likelihood of properly categorizing the MRI scan will decline when significant portions of the MRI are obscured. Hence, larger negative values suggest that the decision process gave more weight to the associated occluded area.

By computing the occlusion map during the prediction using trained models, we verify the MRI scan of the AD patient. This task has been implemented by using Algorithm 3, which is given below. Occlusion sensitivity was calculated by using the visualize.occlusion_sensitivity.OcclusionSensitivity() function of MONAI. It took around 5 h to calculate in Google CoLab Pro + .

**Fig. 13:**
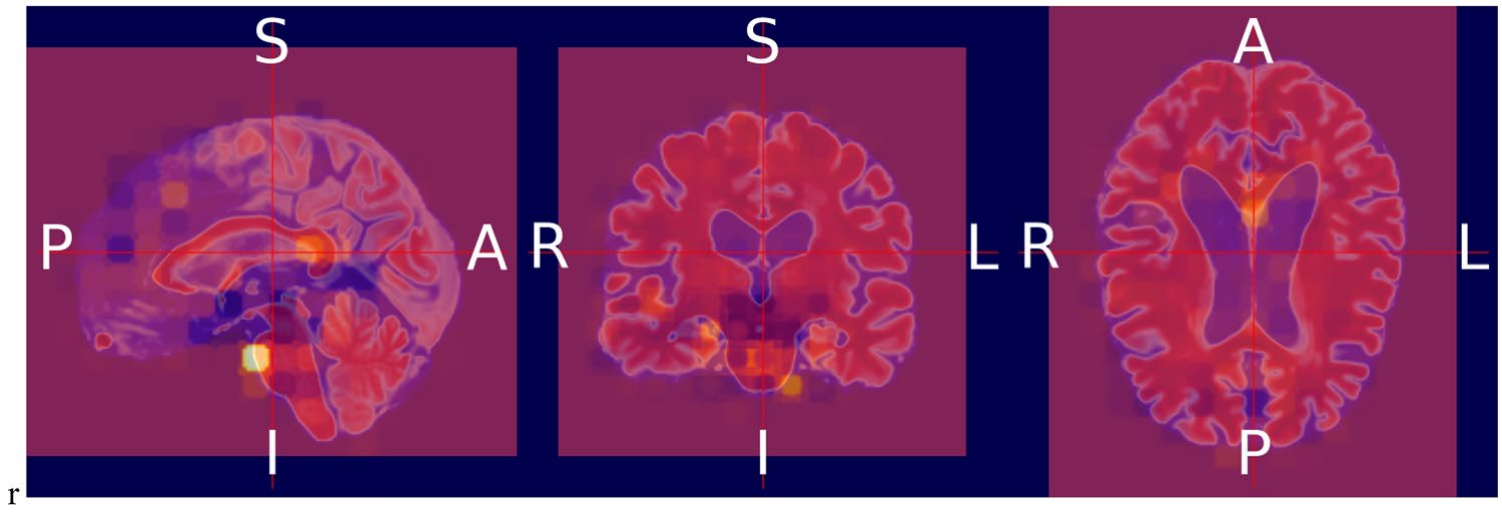
Heat map overlayed on the base MRI scan based on the occlusion sensitivity for the sMCI class [Binary classification model]

**Fig. 14:**
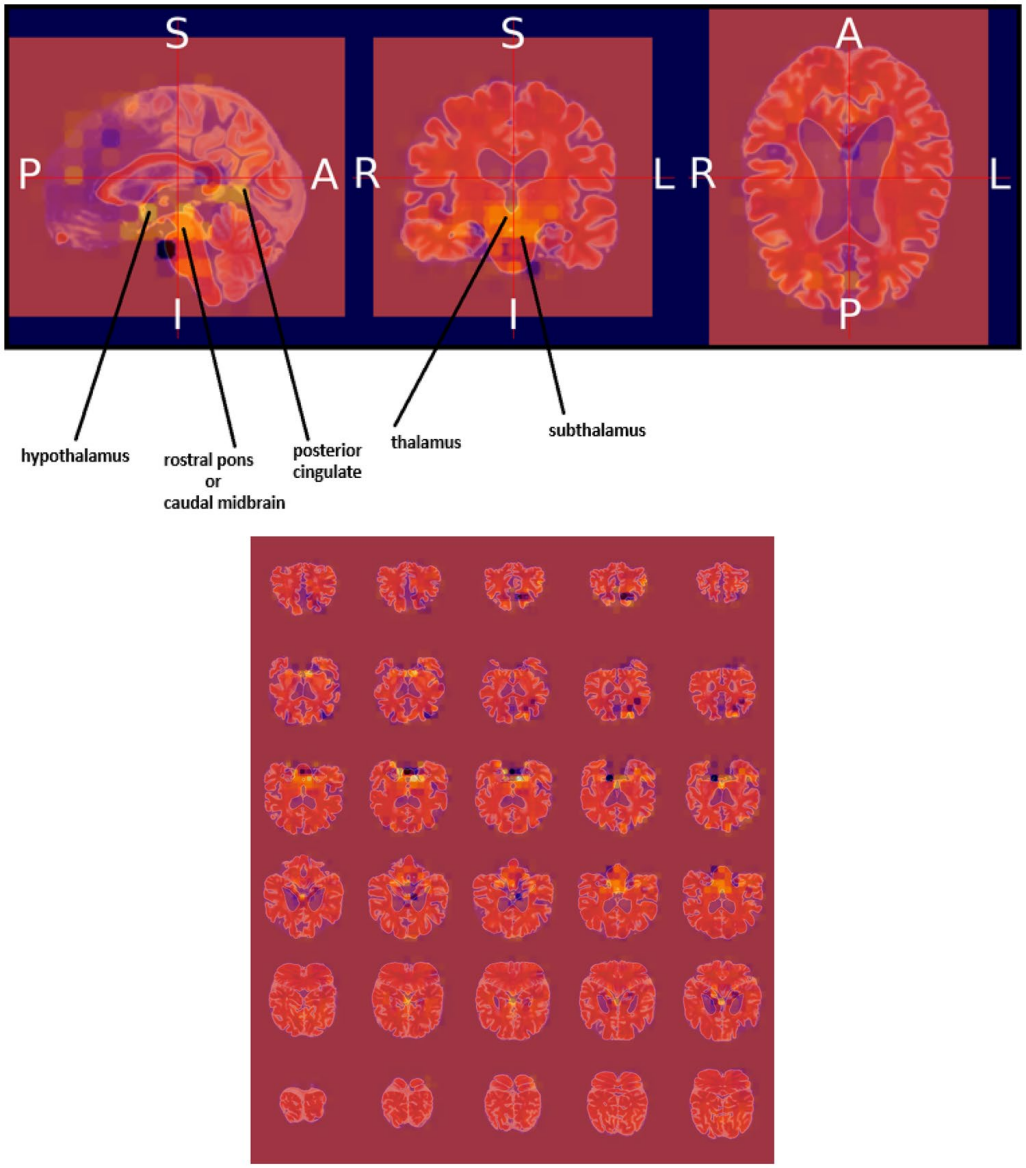
Heat map overlayed on the base MRI scan based on the occlusion sensitivity for the AD class [Binary classification model]

We validate both the models. Figures 13 and 14 illustrate the outcomes of the binary classification model for the sMCI and AD classes, respectively. Compared to Fig. 14, there are less occulated regions in Fig. 13. It verifies that the model's prediction was made accurately (AD). In addition, in Fig. 14, we have highlighted the relevant aspects as determined by neuroradiologist.

**Algorithm 3:**
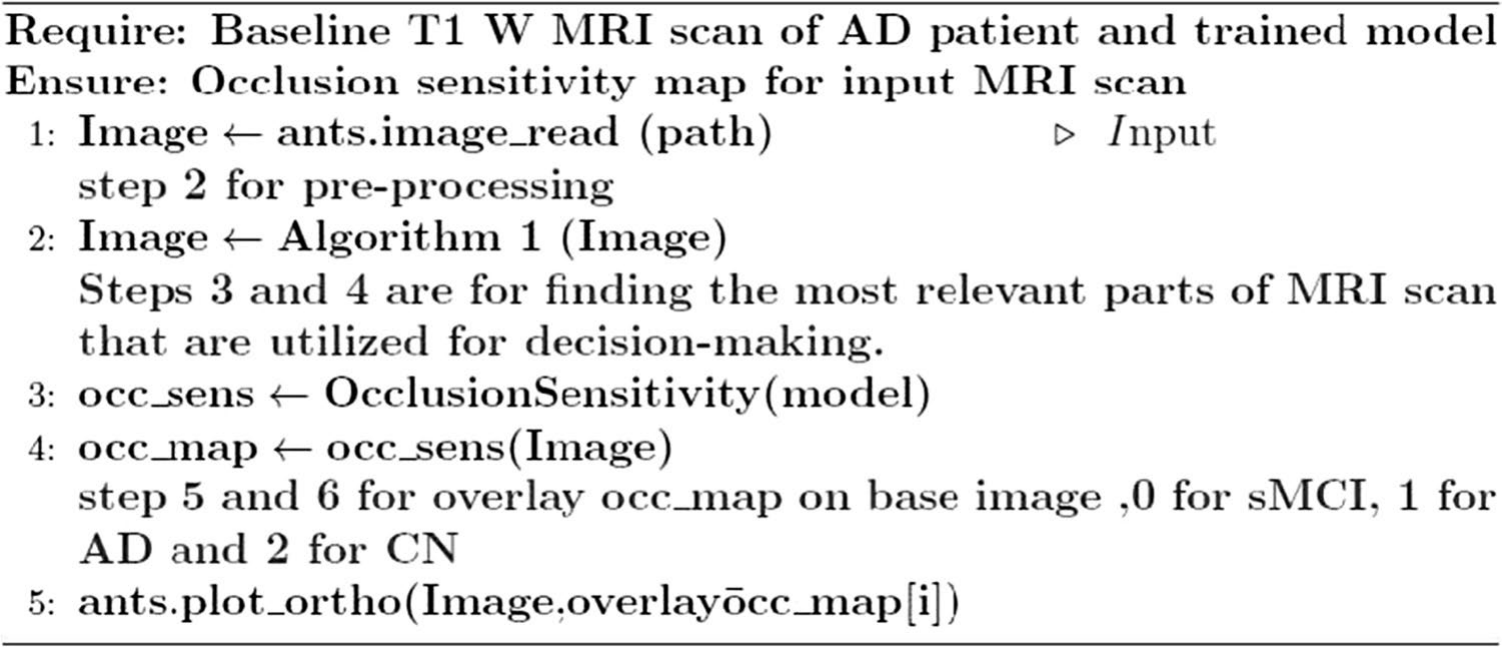
Occlusion sensitivity map

**Fig. 15:**
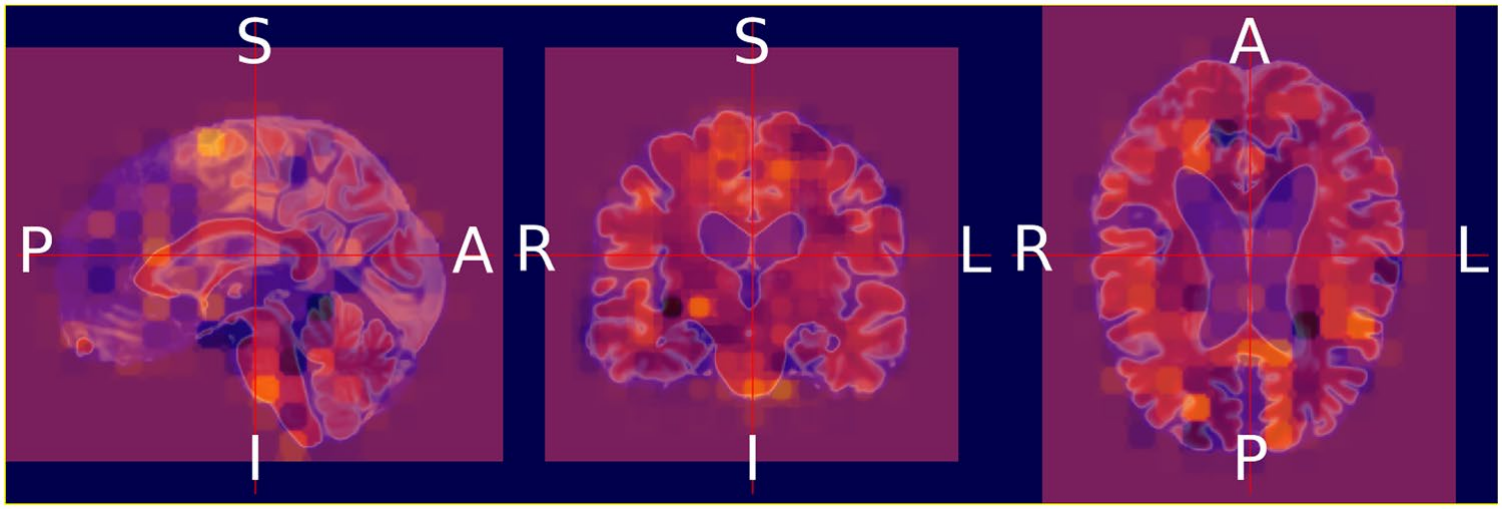
Heat map overlayed on the base MRI scan based on the occlusion sensitivity for the sMCI class [Multi class classification model]

**Fig. 16:**
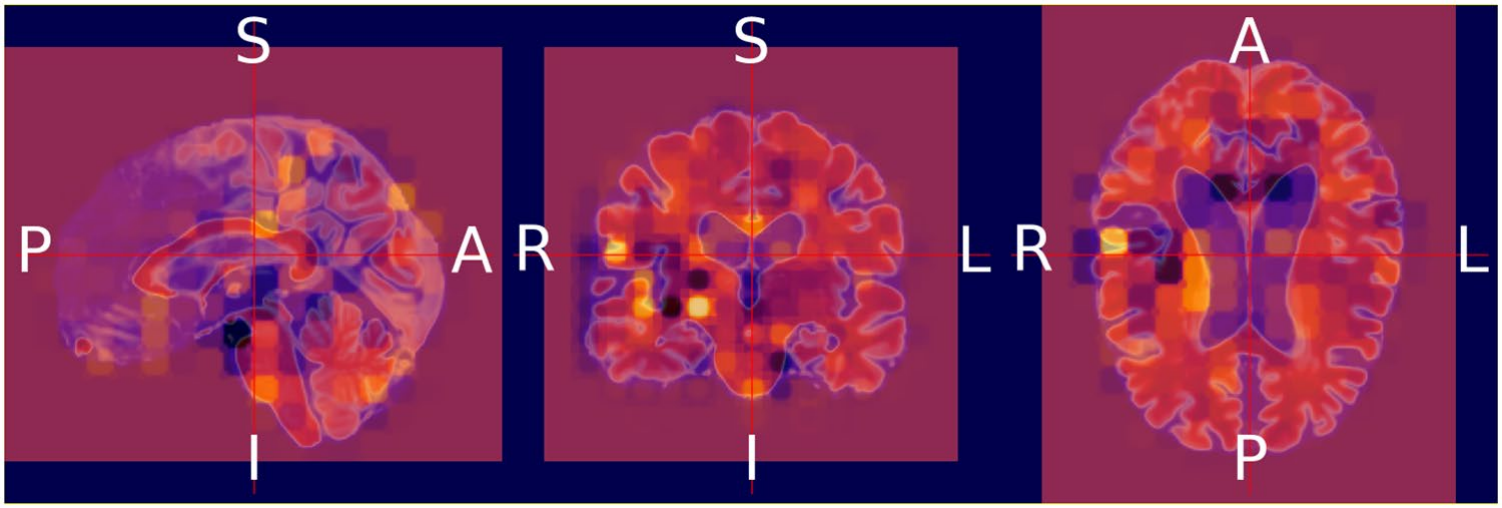
Heat map overlayed on the base MRI scan based on the occlusion sensitivity for the CN class [Multi class classification model]

**Fig. 17:**
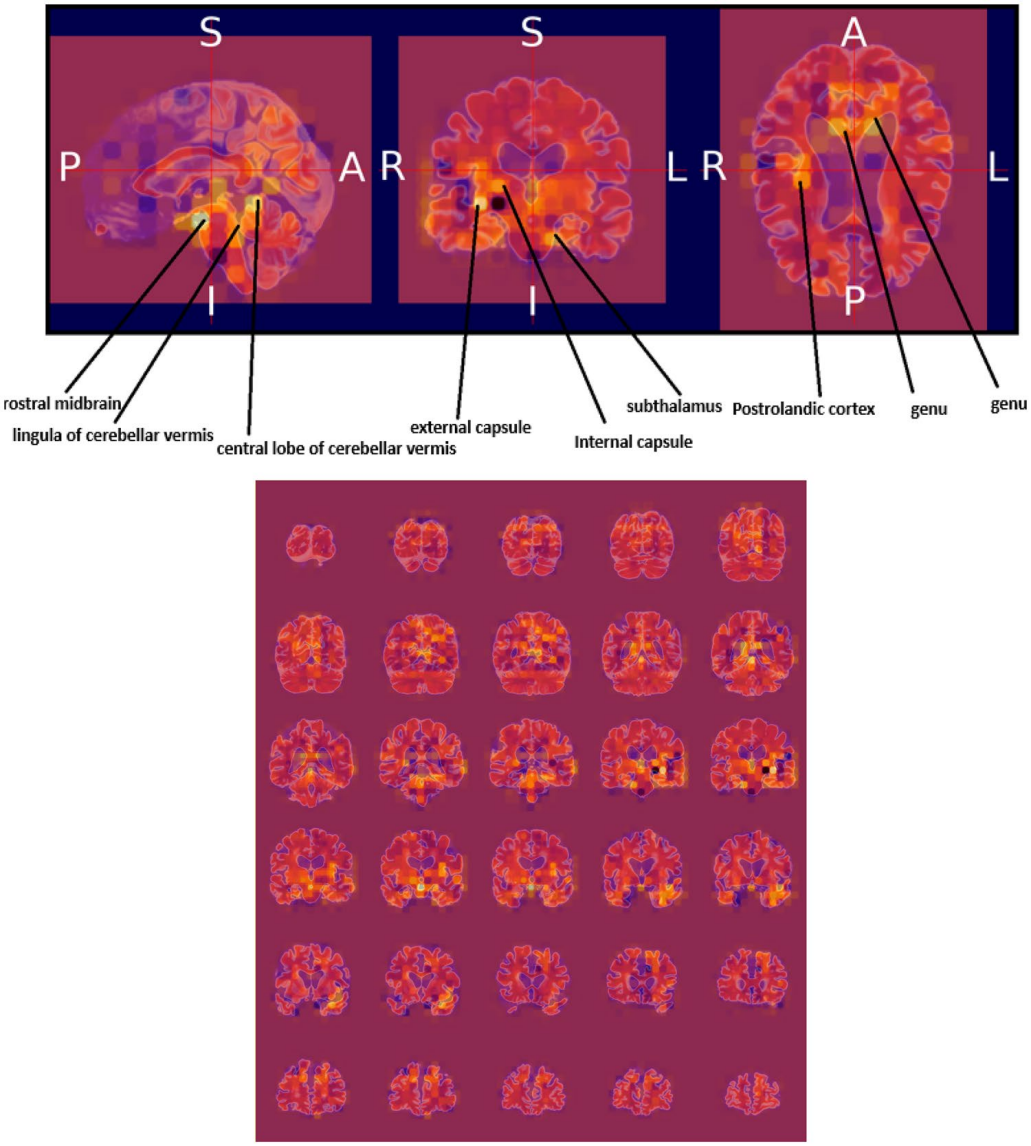
Heat map overlayed on the base MRI scan based on the occlusion sensitivity for the AD class [Multi class classification model]

Figures 15, 16, and 17 illustrate the outcomes of the multi class classification model for the sMCI, CN and AD classes, respectively. Compared to Figs. 15 and 16, there are more occulated regions in Fig. 17. It verifies that the model's prediction was made accurately (AD). In addition, in Fig. 17, we have highlighted the relevant aspects as determined by a neuroradiologist and displayed the 20 slices to make the difference in atrophy clearly noticeable. The link provided in the supplemental material can be utilized to access the scripts developed for this method of creating heatmaps.

### Validation of the binary classification model by using Spanish datasets

To prove the generalizability of the model that has been constructed by employing the novel approach of "fusion of E2EL and TL", we evaluated it for the prediction of the MRI scans of Spanish datasets. This dataset was collected from several HT Medica sites throughout Spain. The dataset includes a diverse community of people with varied degrees of cognitive impairment. The sample population is drawn from a variety of sources, resulting in a representative and diversified sample that reflects the Spanish population. It includes clinical reports and T1W MRI scans from 22 patients with AD and MCI.

The following details are included in reports.

1. Scan date
2. Location of the hospital in Spain
3. Clinical information
4. Findings
5. Conclusion
6. Name of the neuroradiologist with signature and collegiate number.

The clinical information and conclusions were utilized to confirm model predictions by neuroradiologist Dr. M. Alvaro Berbis, Director of R&D and Innovation, HT Médica Madrid, Spain, who is also one of the authors. 19 predictions out of 22 were matched with the clinical findings, yielding an accuracy of 86.36%. The accuracy this model attained using an actual dataset of Spanish patients demonstrates its relevance and generalizability. The results have been shown in Table 2 along with clinical findings and details. Due to privacy issues, Table 2 includes the scan date, results, clinical information, and conclusion only. With the URL provided in the supplemental material, readers may get the Python script used for this validation procedure.

**Table 2:** Results of the validation of binary classification model by using Spanish Datasets

**S.No**	**Id**	**Report date**	**Conclusion**	**Clinical information**	**Model prediction** **(Binary model)**	**Validation by neuroradiologist**
1	1	2/17/2022	Signs of mixed cerebral involution, Incipient periventricular leukoaraiosis	Cognitive impairment of the fronto-temporal profile/Alzheimer’s	sMCI	Yes
2	4	13/11/2021	Moderate corticosubcortical atrophy. We observed mild ventriculomegaly, especially at the expense of the lateral ventricles, probably due to associated subcortical atrophy	Mild-moderate cognitive impairment with amnesic predominance of probable neurodegenerative origin	AD	Yes
3	7	7/9/2021	Signs of mixed cerebral involution associated with foci of gliosis and leukoaraiosis	68 years. Probable mild-moderate Alzheimer's disease	sMCI	Yes
4	13	9/12/2019	Chronic right occipital ischemic infarction. Multiple focal lesions on T2* gradient echo sequence. Mesial temporal atrophy	Acute language deficit after which he presents subacute severe cognitive-behavioral impairment	sMCI	Yes
5	14	23/10/2019	We identified a moderate generalized cortical subcortical atrophy, predominantly posterior parietal with increased furrows at the level of the convexity	Possible Alzheimer	sMCI	No
6	15	6/22/2019	Signs of frontoparietal corticosubcortical atrophy. Atrophy of both hippocampi with frank left predominance. Periventricular leukoaraiosis and multiple ischemic-type lesions in white matter of both hemispheres	Cognitive impairment in a patient with suspected Alzheimer's	sMCI	Yes
7	18	6/9/2018	Bilateral frontotemporal corticosubcortical atrophy as described. Moderate-severe chronic microangiopathy lesions	Cognitive decline	AD	Yes
8	24	5/15/2021	Acute ischemic focus in the left thalamic region.Leukoaraiosis. Foci of gliosis of probable ischemic origin. Signs of involution. Hydrocephalus	Cognitive impairment type Alzheimer. Hydrocephalus	sMCI	Yes
9	47	3/27/2022	Few white matter lesions of chronic small vessel ischemic type. Abnormal atrophy for age with moderate-severe volume loss in both hippocampi, more advanced in the left. Assess primary degenerative process type AD	He does not know what day we are in nor is he able to name the objects on the photo test, she doesn't want to go out	sMCI	Yes
10	48	2/14/2022	GENERALIZED CORTICO-SUBCORTICAL VOLUME LOSS WITH IMPORTANT MESIAL TEMPORARY INVOLVEMENT, ALZHEIMER'S DISEASE CANNOT BE RULED OUT	mild cognitive impairment	sMCI	Yes
11	54	7/11/2021	Atrophy of the described characteristics, assess the possibility of a primary degenerative process of the Alzheimer type	Patient with cognitive impairment suggestive of Alzheimer's disease	sMCI	Yes
12	55	11/2/2021	Mild changes due to chronic cerebrovascular disease. Atrophy of the described characteristics, assess primary degenerative process type AD	Progressive cognitive impairment	sMCI	Yes
13	61	9/20/2021	Changes due to chronic cerebrovascular disease (Fazekas 1–2). Atrophy of the described characteristics, suspicious for an Alzheimer-type degenerative process	A 79-year-old patient with dementia that could be due to Levy bodies or, less likely, Alzheimer's type	sMCI	Yes
14	62	9/14/2021	Moderate signs of diffuse cerebral atrophy with cortical predominance. Mild areas of gliosis and/or demyelination of chronic ischemic origin	Alzheimer's	sMCI	No
15	67	2/12/2020	Small vessel ischemic disease evolved at the supratentorial level. Mild cortico-subcortical involution, somewhat more prominent in the bilateral temporal region	A 66-year-old patient with cognitive impairment suspected of Alzheimer’s but has multiple CVRF. A. familiar’s dementia. Request cranial MRI	sMCI	Yes
16	29	3/10/2022	Possible mild Alzheimer's disease. depressive symptoms	Possible mild Alzheimer's disease. depressive symptoms	AD	Yes
17	37	05/05/2019	Signs of moderate mixed cerebral involution	Alzheimer-type cognitive impairment	sMCI	Yes
18	39	6/10/2018	Periventricular leukoaraiosis and multiple ischemic gliosis-type lesions in the white matter of both hemispheres	Suspected Alzheimer's disease. Assess pattern of atrophy	AD	Yes
19	40	5/23/2018	Isolated Ischemic gliosis-type lesions in the white matter of both hemispheres	Alzheimer's disease with cerebrovascular accident and left hemiparesis. Chronic headache	sMCI	No
20	41	2/9/2022	Calcium deposits with bilateral and symmetrical distribution at the level of the basal ganglia and cerebellar dentate nuclei with non-specific characteristics	A 68-year-old male with suspected progressive cognitive impairment with an amnesic profile. Suspected underlying neurodegenerative process such as Alzheimer's disease. Assess degree of cerebral atrophy	AD	Yes
21	42	26/08/2021	MRI of the skull Signs of mixed cerebral involution associated with foci of ischemic gliosis and leukoaraiosis. Left cerebellar ischemic sequela	Cognitive impairment. Possible Alzheimer's	AD	Yes
22	46	9/5/2018	Severe, generalized cortico-subcortical atrophy of the temporal lobes, suggestive of Alzheimer-type degeneration	Unrelated seizures with two nonspecific lesions in the left semioval center to rule out ischemic or metastatic disease	AD	Yes

## Discussion

We compared our categorization findings to those reported in the literature, as shown in Tables 3 and 4. As converted MCI suggested AD and non-converter MCI showed stable MCI, we also compared the findings for sMCI vs. AD with those for ncMCI vs. cMCI. Most published literature regarding binary classification tasks utilized accuracy, sensitivity (SEN), specificity (SPE), balanced accuracy (BA), and AUC to demonstrate their findings.

**Table 3:** Matching up the findings of the AD vs. SMCI task with the results of the state-of-the-art DL models

**DL model**	**ACC**	**AUC**	**SPE**	**SEN**	**BA**	**Ref**
**3D CNN [EfficientNet-B0]**	**93.10**	**93.00**	**86.20**	**1.00**	**93.10**	*
3D CNN [DenseNet264]	82.50	82.50	82.50	82.50	82.50	[55]
Sparse Regression + 2D CNN	74.82	75.39	78.82	70.93	74.87	[58]
3D CNN	72.5	74.60	82.5	61.0	71.75	[8]
CAE + 3DCNN	73.95	79.11	70.71	77.46	74.08	[19]
3D CNN	86.3	-	88.7	84.0	86.35	[42]
3D CNN	76.0	81.0	76.0	71.0	73.5	[28]
2D CNN	83.81	88.89	87.50	75.76	81.63	[59]
MDNN/2D CNN	81.55	-	73.33	83.83	78.58	[27]
AlexNet + SVM 2D CNN	78.56	-	77.63	91.02	84.32	[23]
MM-SDPN	78.88	-	86.81	68.04	77.42	[60]

* Proposed

**Table 4:** Matching up the findings of the AD vs. CN vs. SMCI task with the results of the state-of-the-art DL models

**DL model**	**ACC**	**AUC**	**Precision**	**Recall**	**F-1 score**	**Ref**
**3D CNN [EfficientNet-B0]**	**87.38**	**91.0**	**86.38**	**87.51**	**86.43**	*
CaffeNet/2D CNN	87.00					[12]
GogleNet/2D CNN	83.20					[12]
3D CNN	64.81	55.5	44.66		41.88	[61]

We evaluated accuracy and AUC during our experiments. By using a confusion matrix of the best fold of the testing, we also computed SEN, SPE, and BA through the following formulas: TP/ (TP + FN), TN/ (TN + FP), and (SEN + SPE)/2, respectively.

The issue of whether patients with MCI can accurately self-diagnose their risk of developing AD remains essential to the development of viable treatments for the disease. Categorizing AD and sMCI is more challenging due to the subtler morphological changes that must be noticed, as demonstrated by the fact that the accuracy of several of the study results included in Table 2 barely reached 70–80%.

The best Level 1 learning model, reported by Suk et al. [60], had a maximum accuracy of 74.82%. It is crucial to highlight that Level 1 approaches alter spatial localization in the feature extraction process of brain imaging data, as they rely on manual feature extraction. Without taking spatial relationships into account, it is hard to guess how the model decides how to classify something in a reliable way. The Level 2 model proposed by Pan et al*.* [59] showed a maximum accuracy of 83.81%. They suggested a multi-view separable pyramid network (MiSePyNet), a 2D CNN model that utilizes 18F-FDG PET images. MiSePyNet was built on the concept of quantized convolution and used independent slice and spatial-wise CNNs for each view. However, this Level 2 research only used a small part of the original datasets, thus disposing of any obvious outliers and making it hard to fairly compare its performance. In another study [42] carried out by Basaia et al*.*, 86.30% accuracy was obtained using a 3D CNN. MRI scans were segmented to create GM, WM, and CSF tissue probability maps in the MNI space. It was also built on a ROI-focused strategy rather than E2EL. Other studies [23, 27, 60] that used 2D TL with a pretrained network or local TL by transferring the knowledge of the AD vs. CN task to predict early diagnosis of AD obtained accuracies up to 82%.Only two research articles regarding multiclass categorization tasks could be found.

One by Wu et al*.* [12] utilized 2D MRI slices and the pre-trained 2D CNN networks CaffeNet and GoogleNet, obtaining an average accuracy of 87.00% and 83.20%, respectively. However, their implementation was based on Level 2 learning, and only obtained a 72.04% (for CaffeNet) and a 67.03% (for GoogleNet) accuracy rate for the classification of sMCI cases. Using Level 3 E2EL,MRI images as input and a basic 3D CNN model, Tufali et al*.* [61] conducted experiments for multiclass classification, but only obtained an average accuracy of 64.33% and an MCI class accuracy of 51.25%.

We achieved an accuracy of 93.10%in the evaluation of unseen data for the binary classification task and 87.38%for the multiclass classification task. This is significantly better than the early AD prediction accuracy reported by state-of-the-art methods in the last five years. Although our models are suitable to use in clinical settings to aid neuroradiologists, further training with more high-quality MRI scans from a diverse range of sources is required to ensure reproducibility.

## Conclusion

Several conclusions can be extracted from the research presented here. Even with neuroimaging, where a limited number of high-dimensional scans are available, the fusion of E2EL with TL allows the obtaining of remarkable results. However, it requires the fine tuning of hyperparameters, and an appropriate 3D CNN architecture specifically designed for TL with excellent potential for generalization; additionally, MRI scans must be thoroughly pre-processed to maintain the spatial link and enhance image quality.

We also observed that MONAI offers a leading framework to implement DL models for medical image analysis, as it is simple to understand and supports a wide range of functions. Additionally, Google Colab Pro + offered the best online cloud-based resources, with access to large RAM and excellent GPUs, thus enabling us to achieve this task despite certain drawbacks such as GPU unavailability under heavy load.

The results obtained in our experiments utilizing the ADNI and IXI datasets demonstrated that our model is more effective and efficient than the current state-of-the-art models for both binary and multiclass tasks. However, there are a number of limitations to this study that need to be addressed in follow-up research.

Furthermore, the model must be implemented in clinical settings where it can be subjected to qualitative examination to determine its robustness. The number of subjects utilized to foster E2EL was still quite low. We anticipate that, as more diverse datasets become available in the future, this approach will lead to more generic learning models.

## Data Availability

The data that underpins the study's conclusions is freely accessible in IXI at https://braindevelopment.org/ixi-dataset/ and in ADNI at https://adni.loni.usc.edu. On reasonable request, the data of the 22 Spanish subjects, which were gathered from several HT Medica sites around Spain and used to validate the model's generalizability, may be provided.

## Appendix

Visit this URL to obtain the supplementary documentation: https://drive.google.com/drive/folders/1MFtlIitvMBnCt34SkCM8ZIMbZ3mn5bNT?usp=share_link

It provide access to the scripts for MRI preprocessing, hyperparameter tuning, and model implementation for both types of tasks, with results, confusion matrices, and AUC graph for every fold. Additionally, the scripts used for generating heatmaps and validating Spanish datasets, can also be accessed.
